# Management of precocious puberty

**DOI:** 10.1186/1687-9856-2013-S1-O10

**Published:** 2013-10-03

**Authors:** Toshiaki Tanaka

**Affiliations:** 1 Tanaka Growth Clinic, Japan

## 

The objectives of treatment for children with central precocious puberty (CPP) are to avoid psychosocial problems caused by early pubertal development and to normalize adult height (AH). A long-acting GnRH analog is the treatment of choice for CPP. GnRH analog administration effectively arrests further development of secondary sex characteristics, slows bone age (BA) maturation, increases pubertal height gain, and is believed to eventually improve AH prognosis. However, the improvement of AH is not well established. It is reported that GnRH analog is effective to improve adult height only in early onset (girls <6 years) CPP [1]. Although BA maturation is decelerated by suppressing gonadotropins with GnRH analog and pubertal period is elongated, growth rate diminishes due to suppressed sex steroid hormone and, in part due to decreased GH secretion.

For the evaluation of efficacy of GnRH analog for adult height improvement, one problem is that the prediction method for adult height in CPP is not established. It is reported that predicted adult height (PAH) using the Bayley-Pinneau table for accelerated BA overestimated AH in untreated patients with CPP, and the PAH based on the projected height SD score for BA is useful [2].

Most Asian countries use a starting dose of 100 µg/kg/month of leuproride acetate depo [3]. During GnRH analog treatment, serum concentrations of LH, testosterone or estradiol should be monitored as well as pubertal changes and bone age, height and height velocity. For some older patients, a dose of up to 180µg/kg/month of leuproride acetate depo is necessary to suppress LH concentration less than 0.5 mIU/ml. When growth velocity is decreased, possible options is to add growth hormone or anabolic steroid hormone only in boys.

The decision to stop therapy should be individualized and based on various factors such as growth velocity, bone age, chronological age, predicted adult height, emotional maturity, and patient’s wish.

After treatment discontinuation, long-term follow up is recommended for adult height, reproductive function and bone mineral density.
